# Proposal for a classification system of radiographic bone changes after cervical disc replacement

**DOI:** 10.1186/s13018-024-04679-y

**Published:** 2024-04-03

**Authors:** Armen Khachatryan, Frank M. Phillips, Todd H. Lanman, Gunnar B. Andersson, Joshua J. Jacobs, Steven M. Kurtz

**Affiliations:** 1 The Disc Replacement Center, Salt Lake City, UT USA; 2 Rush University Medical Center, Chicago, IL USA; 3 Lanman Spinal Neurosurgery, Los Angeles, CA USA; 4 Implant Research Core, School of Biomedical Science, Engineering, and Health Systems, Drexel University, Philadelphia, PA USA

**Keywords:** Cervical total disc replacement, Osteolysis, Bone loss, Classification

## Abstract

**Background:**

The goal of this study is to propose a classification system with a common nomenclature for radiographic observations of periprosthetic bone changes following cTDR.

**Methods:**

Aided by serial plain radiographs from recent cTDR cases (34 patients; 44 devices), a panel of experts assembled for the purpose of creating a classification system to aid in reproducibly and accurately identifying bony changes and assessing cTDR radiographic appearance. Subdividing the superior and inferior vertebral bodies into 3 equal sections, observed bone loss such as endplate rounding, cystic erosion adjacent to the endplate, and cystic erosion not adjacent to the endplate, is recorded. Determining if bone loss is progressive, based on serial radiographs, and estimating severity of bone loss (measured by the percentage of end plate involved) is recorded. Additional relevant bony changes and device observations include radiolucent lines, heterotopic ossification, vertebral body olisthesis, loss of core implant height, and presence of device migration, and subsidence.

**Results:**

Serial radiographs from 19 patients (25 devices) implanted with a variety of cTDR designs were assessed by 6 investigators including clinicians and scientists experienced in cTDR or appendicular skeleton joint replacement. The overall agreement of assessments ranged from 49.9% (95% bootstrap confidence interval 45.1–73.1%) to 94.7% (95% CI 86.9–100.0%). There was reasonable agreement on the presence or absence of bone loss or radiolucencies (range: 58.4% (95% CI 51.5–82.7%) to 94.7% (95% CI 86.9–100.0%), as well as in the progression of radiolucent lines (82.9% (95% CI 74.4–96.5%)).

**Conclusions:**

The novel classification system proposed demonstrated good concordance among experienced investigators in this field and represents a useful advancement for improving reporting in cTDR studies.

**Supplementary Information:**

The online version contains supplementary material available at 10.1186/s13018-024-04679-y.

## Introduction

Cervical total disc replacement (cTDR) is becoming an established alternative to fusion for treatment of degenerative disc disease and associated radiculopathy and myelopathy [1–4]. Starting in the 2000s, the clinical effectiveness of cTDR has been supported by randomized clinical trials, now with intermediate- and long-term follow-up [2, 4–10]. Early cTDR designs were often based on traditional orthopaedic biomaterials, including polyethylene, CoCr alloys and Ti alloys [3, 10], but the lower biomechanical loading demands of the cervical spine relative to the lumbar spine have also encouraged innovative designers to investigate new bearing materials [11], incorporating metallic alloys, ceramics, polycarbonate urethane, and/or PEEK, with no previous clinical precedent as bearing materials in large total joint replacements. As cTDR designs and its biomaterials continue to evolve, and as utilization of the procedure increases with longer historical exposure, there has been increased attention on identifying mechanisms of cTDR failure [12], such as subsidence, migration, and/or wear, along with recommended treatment paradigms for each clinical failure scenario [12]. Ideally, classification and treatment pathways should be generalized with the understanding that cTDR failures, and their revision approaches, may be design specific.

Since the beginning of large-joint arthroplasty, clinicians and orthopedic researchers have used radiographs to evaluate the status of implant fixation and the likelihood of impending revision surgery. For example, Delee and Charnley [13] and Gruen [14] proposed acetabular and femoral zones in the 1970s which later came to be widely used in describing the location and progression of radiolucent lines and osteolytic lesions around a total hip replacement. Progressive radiolucent lines around a hip component of greater than 2 mm have been found to be associated with clinically relevant loosening that may require revision [13, 15]. However, an equivalent, generally accepted radiographic classification and treatment approach is not yet available in cTDR, in part because of the recent adoption of this relatively new procedure, and also because of the diversity in implant designs. The clinical situation is more complex in cTDR than in large joints, in which diagnosis and treatment are generally based on radiographic examination as the “keystone” diagnostic tool [15]. By contrast, cTDR treatment decisions involving revision are less frequently undertaken based on radiographs alone, without further workup that may include computed tomography (CT) scans and/or magnetic resonance imaging (MRI). Of these imaging modalities, CT is the most reliable test for bone loss.

Nevertheless, plain radiographic assessment remains the front-line patient assessment tool for cTDR [12, 16]. Previous studies have underscored the need for classification of radiographic changes around cTDR including heterotopic ossification (HO) [17, 18] and bone loss [12, 19]. Bone loss around orthopedic implants can result from sepsis [20, 21], or aseptically due to bone adaptation or remodeling due to stress shielding (sometimes referred to as “Wolff’s law”) [22], osteoporosis [23], fluid pressure [24], as well as the well-described chronic inflammatory reaction to wear debris termed “osteolysis” in peripheral joint arthroplasty [19]. Today, the etiology of bone loss around large-joint implants can be effectively elucidated with plain radiographs and long-term clinical experience with specific designs [15], but whether a radiographic bone lesion is septic or aseptic, or due to bone adaptation or particulate wear debris, may require confirmation by pathologic examination of retrieved periprosthetic tissues [25] as well as microbiological cultures of synovial fluid and periprosthetic tissues.

However, authors in the spine field often use terms such as wear, osteolysis, and bone loss interchangeably to describe radiographic changes around cTDRs, without pathologic confirmation for the etiology of bone loss [19, 26, 27]. Zavras et al. [12] have recently proposed a cTDR failure classification system, with wear as a type of failure with subclassification of osteolysis with minimal or severe bone loss. The ambiguity and lack of specificity in the radiographic assessment of periprosthetic bony changes around cTDR is a barrier to effective scientific communication and the development of effective treatment recommendations.

Consequently, the purpose of the present study was to develop and assess a radiographic classification system for reactive changes after cTDR. We sought to answer the following principal research question: can plain radiographs be used for accurate and repeatable classification of bony changes after cTDR? To answer this question, our team developed and evaluated a classification system using blinded and de-identified radiographic case studies drawn from their clinical experience with multiple implant designs.

## Methods

### Development of the classification system

A classification system in the present study was iteratively developed over a 12-month period, during which the co-authors convened as a panel to collaboratively evaluate serial radiographs of cTDRs and previous classification studies for cTDR [12, 18, 19, 28] and total joint replacement [13, 14]. The panel consisted of six investigators, including four experienced cTDR surgeons (AK, FP, TL, GA), an experienced large joint orthopaedic surgeon (JJ), and a clinical researcher (SMK) with experience in TDR revision and assessment of bony changes in radiographs around large total joint replacements. Because our goal was to assess radiographic changes over time, and cTDRs routinely exhibit gaps or radiolucent lines immediately after implantation that gradually fill in over time, assessments were not made based on an isolated set of radiographs. This approach was consistent with best practices of assessing bony changes around hip and knee total joints. We initially considered the use of anterior–posterior (AP) radiographs as part of the classification system, however we found that the APs of the cTDR cases available to us were more variable in terms of readability and angle of the incident beam, making evaluation of the interface more difficult. Hence, we relied on lateral radiographs as the basis for our classification system. Radiographs from 34 patients representing 44 implants from 7 implant systems were reviewed to aid in the development of the classification system, specifically for the purpose of identifying and agreeing on bone change descriptions and important implant considerations. The radiographs were sourced from the spine surgeon coauthors’ recent cTDR cases, in particular those with exemplary imaging.

**Fig. 1:**
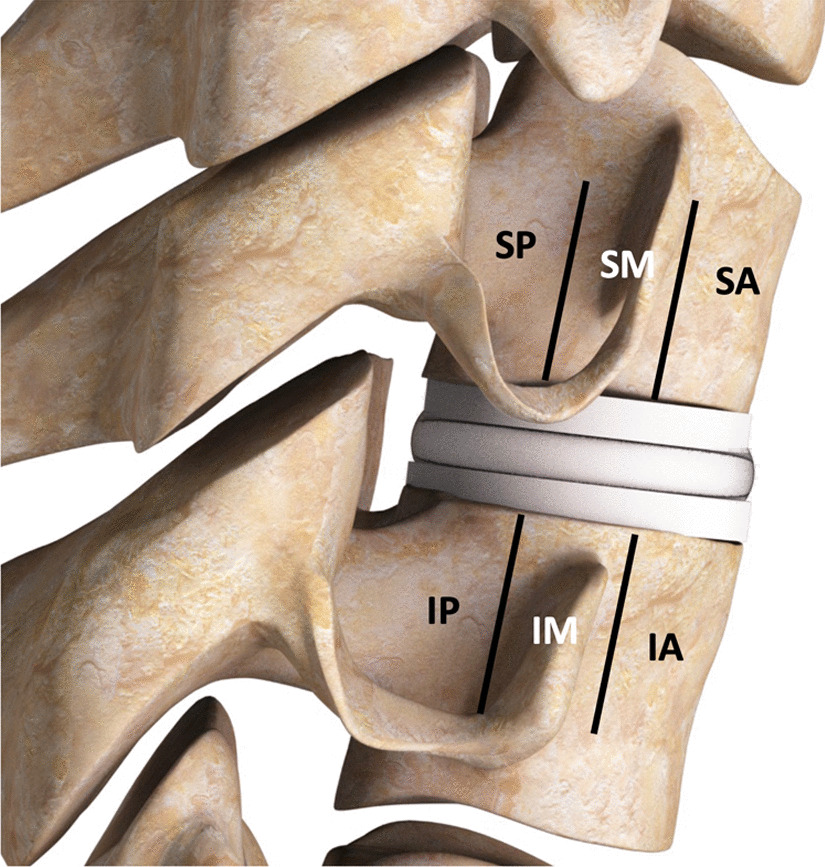
Schematic of six periprosthetic regions for assessment of bony changes after cTDR based on lateral radiographs: superior posterior (SP), superior middle (SM), superior anterior (SA), inferior posterior (IP), inferior middle (IM), inferior anterior (IA)

The classification protocol for assessing radiographic changes was based on lateral plain radiographs of the cervical spine, dividing the vertebral body into three sections, anterior, middle, posterior, approximating sections as shown in Fig. 1. Attention was focused on characterizing changes around both the superior and inferior endplates in these three sections. Note that these figures are artists renderings of radiographs to convey the particular observed anatomy. Example of radiographs from patients used in the study are included in the Additional file 1.

**Fig. 2:**
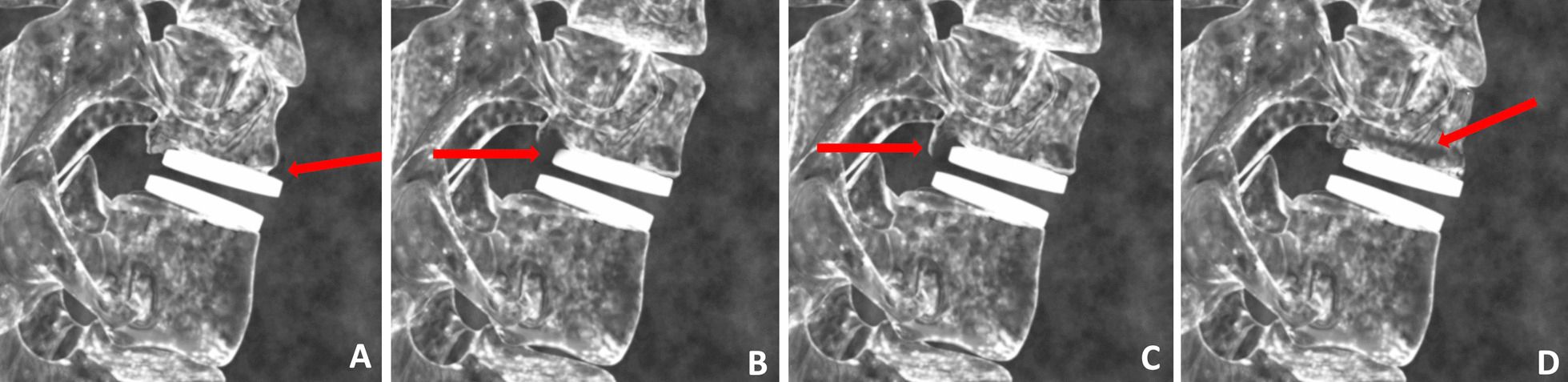
Bony changes of potential clinical relevance around cTDR: **A** Endplate Rounding; **B** Cystic Erosion Adjacent to Endplate w/Diffuse Margin; **C** Cystic Erosion Adjacent to Endplate w/Sclerotic Margin; **D** Cystic Bone Loss Not Adjacent to the Endplate

Bony changes of potential clinical relevance may include:

1. Endplate Rounding (Fig. 2a). This type of bony change has previously been observed around many types of implant designs [28, 29].
2. Cystic Erosion Adjacent to Endplate w/ Diffuse Margin. This type of change can manifest as a radiolucent shadow with poorly defined border (Fig. 2b).
3. Cystic Erosion Adjacent to Endplate w/ Sclerotic Margin. This manifests as a radiolucent shadow with defined border of radiodense bone (Fig. 2c).
4. Cystic Bone Loss Not Adjacent to the Endplate (Fig. 2d).

The severity of the bone loss (i.e., the degree to which the endplates are unsupported) was assessed using the following characterization scheme:

1. None = Neither endplate exhibits bone loss
2. Mild = If either endplate exhibits bone loss that includes an unsupported portion of the endplate that is less than 1/6th of the endplate
3. Moderate = If either endplate exhibits bone loss that includes an unsupported portion of the endplate that is between 1/6th and 1/3rd of the endplate
4. Severe = If either endplate exhibits bone loss that includes an unsupported portion of the endplate that is more than 1/3rd of the endplate

Note that these changes were recorded only if they were not apparent on the immediate postoperative X-rays. Serial radiographs were required to judge the presence and severity of progression. Progression was recorded if the severity of the bony changes (as defined above) increased between the two most recent follow up radiographic series. If there is only one follow up radiographic series after the immediate postoperative films, the determination of progression is far less meaningful; it has been our observation that bony changes up to two years postoperatively may stabilize, showing no further progression on further follow up, up to 5 years.

We also developed a classification scheme to identify the location of radiolucent lines and noted if they were progressive. In the hip and knee literature, radiolucent lines of greater than 2 mm are generally considered to be indicative of implant loosening, however utility of assessing radiolucent lines at the bone implant interface of cTDR is less well defined. Therefore, based on the cTDR images in the present study, we identified the presence of radiolucent lines at the bone implant interface, regardless of their thickness.

Heterotopic ossification (HO) is frequently observed after cTDR and therefore included in the present study. In each of the assessment regions, we identified HO formation on the most recent radiograph based on McAfee classification system [18]:

1. Class 0—No HO present
2. Class I—Presence of HO in front of vertebral body but not in the anatomic disc space
3. Class II—Presence of HO in the disc space, possible affecting the prosthesis’s function.
4. Class III—Bridging HO with prosthesis’s motion still preserved.
5. Class IV—Complete fusion of the segment with absence of motion in flexion/extension.

We also characterized the changes to the implant and its intervertebral positioning. Core implant height is commonly used to assess the performance of the implant over its lifetime by comparing serial radiographs over time, looking for changes in the height of the implant. Core implant height changes were classified as either none/minor (< 50% height loss) or moderate/collapse (> 50% height loss). Similarly, migration, subsidence, and distortion of the core with olisthesis were noted (yes/no). Migration and subsidence can be difficult to quantify. Some hip replacement studies have used 3 mm as a threshold [30], but in cervical application, this is less defined. Therefore, these were noted as either present or not (yes/no), based on the available information of the case. Migration was defined as anterior/posterior/medial/lateral movement of implant relative to vertebral body as compared between immediate post-op and most current radiograph. Subsidence was defined as superior/inferior movement of implant relative to vertebral body as compared between immediate post-op and most current radiograph. Olisthesis was used to describe translation of superior endplate of well-fixed implants relative to the inferior endplate. Spondylolisthesis was used to describe translation of the superior endplate anterior with respect to the inferior endplate. Retrolisthesis was used to describe translation of the superior endplate posterior with respect to the inferior endplate. As with all radiographic assessment and to use the proposed classification system effectively, the importance of good quality, well positioned, serial radiographs for assessment is very important, as this can affect the ability to assess the bony changes.

**Fig. 3:**
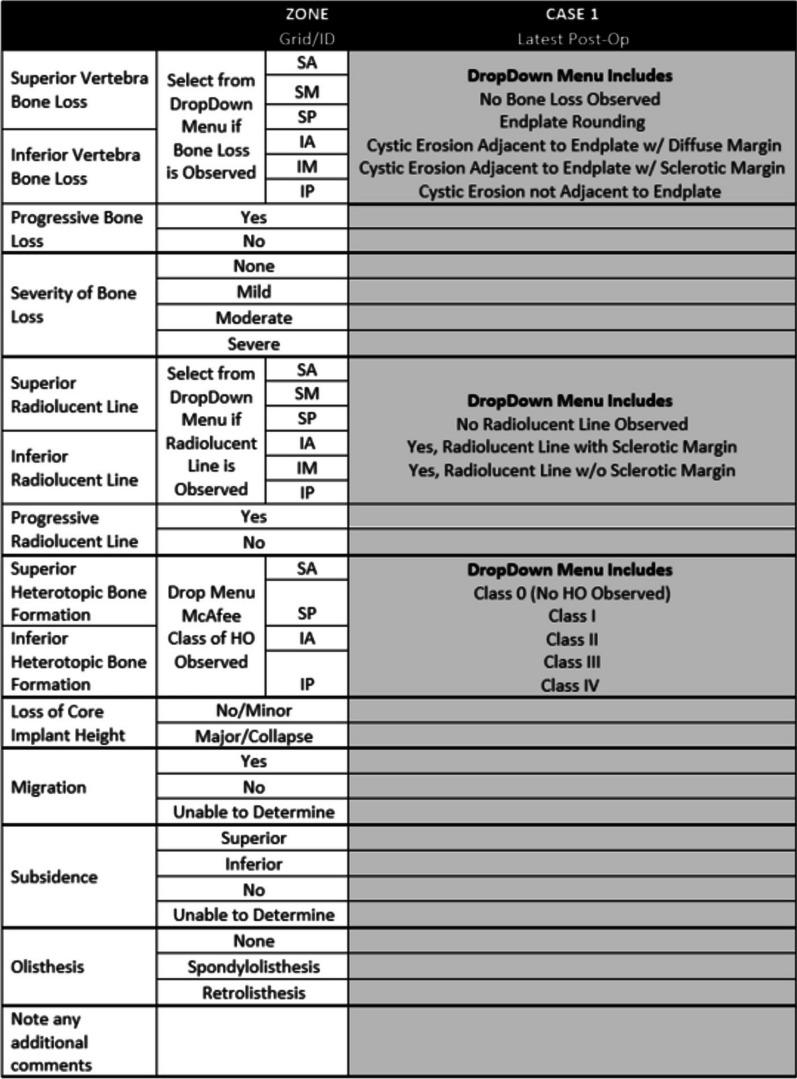
Case report form used to collect data

**Fig. 4:**
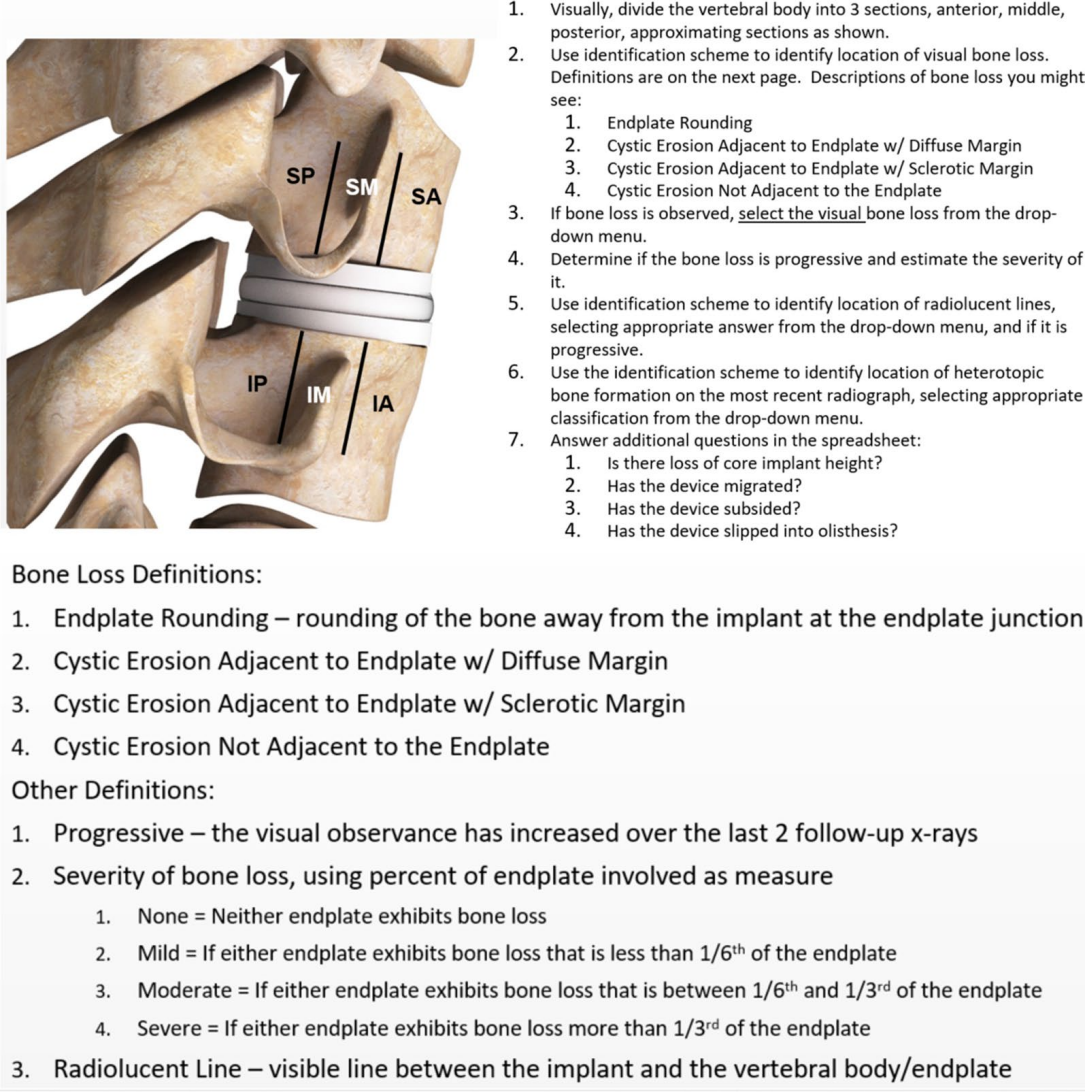
Protocol used for assessment of bone loss from radiographs

In developing the classification system, several iterations of general protocol and data collection form were generated. Figures 3 and 4 detail the protocol provided to the investigators as well as the form used to collect their observations.

### Gathering the data

The case series for the present study was an assembly of serial radiographs from cTDR cases by the spine surgeon investigators. The study protocol was reviewed by an external IRB and found to be IRB exempt.

For the present study, the serial radiographs from 19 patients with 25 implants were assembled and assessed using the classification system. The case series was assembled based on historical cases from the 4 spine surgeon investigators, and because radiographic changes were the focus of the assessment, inclusion of cases was based on availability of serial radiographs. For a case to be included, radiographs from pre-operative, post-operative, and latest follow-up timepoints had to be available, along with clinical presentation information. The pre-operative radiograph was crucial for understanding existing bone condition/anomalies prior to implant-related bone changes, and utilizing cases from a variety of manufacturers and designs helped reduce bias and focus on bone changes. Specific implantation duration was not required. The cases included the following implant systems: Discover (n = 1 patient, DePuy Spine), ProDisc-C (n = 2; Centinel Spine), Simplify (n = 2; NuVasive), M6-C (n = 5; Orthofix), Prestige LP (n = 2; Medtronic), Mobi-C (n = 9; ZimVie), and PCM (n = 4, NuVasive). There were 14 single-level patients, 4 two-level patients, and 1 three-level patient included as part of the study.

To simulate clinical situation as closely as possible, in evaluating case series, panel members considered patient reported symptoms throughout the course of their treatment and serial radiographs typically including index surgery, one-, two-, and five-years post-op. The 6 investigators were provided de-identified serial radiographs and clinical information for each case in the series. The clinical symptoms were used by the investigators to consider potential issues that might be observed in the radiographs, such as common bone loss scenarios, implant performance, potential bony abnormalities, or unique radiographic findings. This information was captured in the notes section of the data collection form (Fig. 4). Independently, each investigator reviewed each case according to the protocol, and provided observations using the data collection form. The data was then pooled, and statistical analyses conducted.

### Statistical methods

The concordance between investigators was found by multiple pairwise comparison of investigator assessments. The maximum number of pairwise comparisons was 375. Concordance was determined by the number of paired observations, as compared with the total number of possible paired comparisons among investigators. Thus, if all of the paired comparisons in each of the region were consistent, that would result in 100% concordance or agreement (375 matching assessments out of a maximum 375 total possible assessments).

**Fig. 5:**
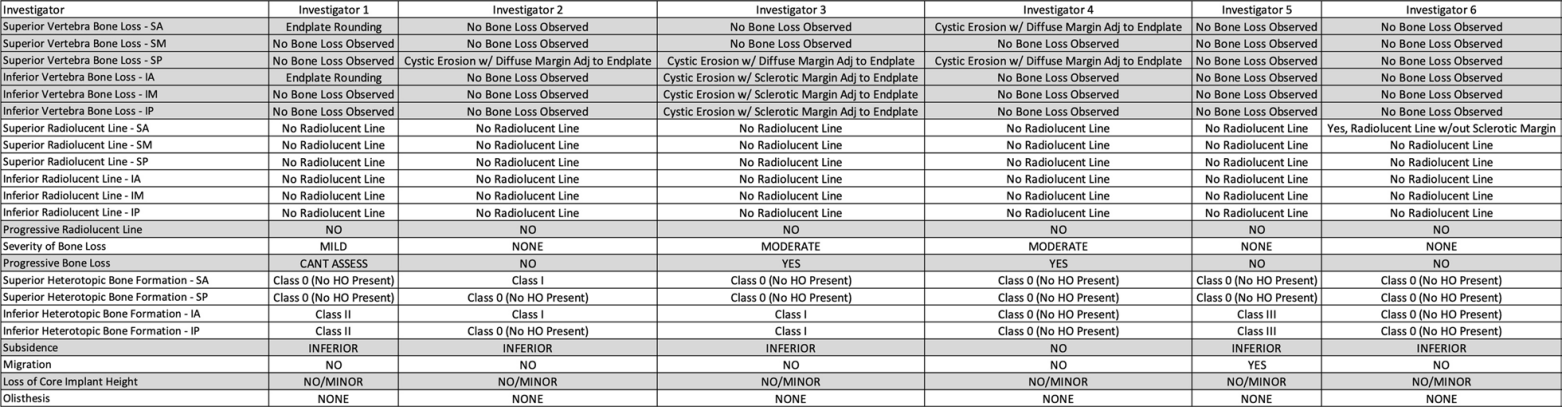
Example of case assessment data from six investigators

A random effects model was hypothesized as the data generating mechanism in order to evaluate the uncertainty in the estimates of overall agreement for each measurement. The model assumed that the 6 raters and 25 subjects were selected at random from populations of raters and subjects. A bootstrapping approach [31] was used to determine empirical sampling distributions for overall agreement. This was accomplished by creating 500 bootstrap samples by sampling 6 raters from the set of available raters with replacement and sampling 25 subjects from the set of available subjects with replacement; and then determining overall agreement as usual for each sample. The lower and upper bounds of the 95% confidence intervals were determined non-parametrically as the 2.5th and 97.5th percentile values. Agreement values smaller than lower bounds of the confidence intervals may be statistically ruled out. Overall agreement may be more interpretable in many cases than Kappa or Krippendorff’s Alpha values, which are very similar, are chance adjusted agreement rates but depend highly on prevalence of individual findings and marginal distributions (Fig. 5).

## Results

The overall agreement of assessments ranged from 49.9% (45.1–73.1%) to 94.7% (86.9–100.0%) (Table 1). There was reasonable agreement on the presence or absence of bone loss or radiolucencies (range: 58.4 (51.5–82.7%) to 94.7% (86.9–100.0%), Table 1), as well as in the progression of radiolucent lines (82.9% (74.4–96.5%)). However, the lowest agreement was found on the assessment of severity of bone loss (49.9% (45.1–73.1%)) or the progression of bone loss (66.2% (59.7–87.7%)). High overall agreement was achieved in binary assessments of HO (range: 75.2 (66.9–91.5%) to 91.7% (83.2–100.0%)) and implant function, such as the presence of subsidence (82.2% (73.1–94.9%)), migration (90.3% (83.2–100.0%)), loss of core implant height (90.9% (83.2–100.0%)), and olisthesis (72.0% (64.8–91.7%)).

**Table 1:** Summary of agreement in radiographic assessments in proposed classification system

Variable	Agreement pairs	Total pairs	Overall agreement (%)	95% LB	95% UB	90% LB	90% UB
Superior vertebra bone loss—SA	219	375	58.4	51.5	82.7	53.5	80.0
Superior vertebra bone loss—SM	353	375	94.1	86.9	100.0	88.7	100.0
Superior vertebra bone loss—SP	297	375	79.2	70.4	95.5	72.4	92.9
Inferior vertebra bone loss—IA	229	375	61.1	52.5	86.1	54.9	83.1
Inferior vertebra bone loss—IM	355	375	94.7	86.9	100.0	88.4	100.0
Inferior vertebra bone loss—IP	350	375	93.3	85.1	100.0	86.7	100.0
Superior radiolucent line—SA	274	375	73.1	61.3	91.2	63.2	88.9
Superior radiolucent line—SM	302	375	80.5	69.9	95.2	71.7	93.9
Superior radiolucent line—SP	284	375	75.7	67.2	93.1	68.5	91.5
Inferior radiolucent line—IA	323	375	86.1	78.1	97.3	79.9	96.3
Inferior radiolucent line—IM	334	375	89.1	79.7	100.0	81.3	98.7
Inferior radiolucent line—IP	335	375	89.3	81.6	100.0	83.2	98.7
Progressive radiolucent line	311	375	82.9	74.4	96.5	75.9	95.3
Severity of bone loss	187	375	49.9	45.1	73.1	46.7	70.4
Progressive bone loss	245	370	66.2	59.7	87.7	62.0	83.7
Superior heterotopic bone formation—SA	317	375	84.5	75.7	96.5	77.9	94.8
Superior heterotopic bone formation—SP	282	375	75.2	66.9	91.5	69.5	90.1
Inferior heterotopic bone formation—IA	344	375	91.7	83.2	100.0	84.8	100.0
Inferior heterotopic bone formation—IP	335	375	89.3	81.3	98.7	82.9	98.7
Subsidence	291	355	82.0	73.1	94.9	75.1	93.7
Migration	325	360	90.3	83.2	100.0	85.1	97.9
Loss of core implant height	341	375	90.9	83.2	100.0	85.1	98.7
Olisthesis	270	375	72.0	64.8	91.7	65.9	89.7

*LB* Lower bound of confidence interval; *UB* Upper bound of confidence interval

## Discussion

As the usage of cervical arthroplasty has increased over the past 20 years, the prevalence of clinical and device-related failures associated with this technology has also increased, motivating the present study to develop a clinically useful classification system to aide clinicians with assessing bony changes over time. In the present study, we developed a robust classification scheme for bony changes around cTDR that was evaluated across a broad range of contemporary implant designs. We utilized serial radiographs of routine clinical cases in our radiographic assessment of cTDR. It became increasingly clear during our collaboration how important serial radiographs are in accurately assessing any progression of bone loss. We routinely found evidence of bone-implant gaps upon assessment of post-operative radiographs that did not fill in or remodel up to five years of implantation. Without access to serial radiographs, those initial unresolved bone-implant gaps could be misinterpreted as bone loss. Therefore, if only a single radiographic observation is available, clinical interpretation is problematic, and additional imaging studies, including computed tomography (CT) and magnetic resonance imaging (MRI) are strongly recommended prior to undertaking further clinical intervention.

There are relatively few studies that have addressed classification of cTDR failure modes that focus specifically on the bone-implant interface [12]. Zavras et al. [12] proposed a general classification for cTDR reasons for revision, that specifically focused on failures that required revision surgery. Zavras and colleagues also classified revision due to septic loosening as a sub classification of infection, whereas revisions due to implant wear were subclassified as either with or without osteolysis. The implication of Zavras’ classification system is that osteolysis is solely associated with wear-related failure of cTDR. We certainly agree with Zavras’ identification of wear as a potential mechanism for osteolysis in the spine, however given that other mechanisms can result in periprosthetic bone loss, such as infection and stress-shielding, we underscore the importance of classifying bone loss based on imaging without attribution of etiology until histopathological confirmation of the root cause has been determined [19]. In large joint orthopaedics, decades of early fixation methods and historical polyethylene bearing materials resulted in radiographic classification systems for bone loss that reflected a time when particulate wear debris from bone cement, metal, and polyethylene was responsible for many more clinical failures than infection. A similar situation does not occur today with cTDR, in which wear related revisions and infection as documented in prospective randomized clinical trials [2, 5–9], are both equally low in incidence, and hence the etiology of periprosthetic bone loss associated with cTDR cannot be reliably assumed based on radiographs alone. In hip and knee replacements, clinical experience has evolved over decades to educate the intuition of surgeons with the radiographic interpretation of bone loss due to non-inflammatory bone adaptation in comparison with inflammatory mechanisms of osteolysis such as infection and particulate wear debris [19]. However, this is not the case with cTDR in which the clinical experience with radiographic interpretation of periprosthetic bony changes is still relatively early in its development. Although osteolysis around cTDR has been raised as a potential clinical concern [19, 27], the use of a reliable radiographic classification system as proposed here, coupled with device and tissue retrieval analysis, should improve future scientific communications about bone loss around total disc replacements, along with their reasons for revision.

Radiographic outcomes are publicly reported for cTDR designs that have completed clinical trials as part of their regulatory approval process by the United States Food and Drug Administration (FDA) (https://www.accessdata.fda.gov/scripts/cdrh/cfdocs/cfpma/pma.cfm). Radiographic outcomes and observations were extracted from the summaries of safety and effectiveness (SSED) from 12 FDA-approved cTDRs currently on the US market and summarized in Table 2. Overall, these findings included assessments of heterotopic ossification, radiolucencies, disc height, migration, subsidence, and loosening (Table 2). However, the reports lacked assessments of bone loss, and a range of methodologies was used by sponsors in radiographic assessments of cTDR, highlighting the need for standardization in this area. Similar findings have been reported for longer-term radiographic studies: a recent systematic review and meta-analysis aggregated all available 5 + year clinical outcomes for cTDR and reported that observations of bone loss/osteolysis were largely absent (or perhaps reported under other terminology or conditions) [32]. Only one reviewed study identified osteolysis, which was reported for six patients out of 715 [33]. Similarly, a recent analysis of shorter-term results found a low number of studies reporting osteolysis [34], and a review regarding cTDR radiological outcomes including HO lacked guidance on osteolysis/bone loss appearance [35]. While reports of radiolucencies were more common in the SSEDs and clinical trials, explanation of the assessment criteria used were often lacking. In our assessment, we considered progression of radiolucencies to be an additional and more telling indicator of the bone-implant interface. Thus, our study adds to the body of clinical findings by highlighting a bone loss metric that was perhaps poorly understood when these trials were designed and warrants continued research even today.

**Table 2:** SSED summary

Device SSED	IDE # of patients	Follow-up	Heterotopic ossification classification type	Radiolucencies	Disc height measurement	Migration/subsidence/loosening	Radiographic success
Prestige (one-level) July 2007	276	6 weeks, 3-, 6-, 12-, 24-months and annually thereafter	NR	NR	Assessment criteria: maintenance of height (height change ≤ 2 mm from 6-week post-op height) Success at 24 mos: 90/92 (97.8%)	Assessment criteria: NR Displacement/Loosening (reported as AE): 2/276 patients (0.7%), 2 events Subsidence (reported as AE): 1/276 patients (0.4%), 1 event	Assessment criteria: (I) the existence of flexion/extension angular motion in a range of > 4° to ≤ 20° and 2) no evidence of bridging trabecular bone forming a continuous connection between vertebral bodies Success at 24 mos: 85/117 (72.6%)
Prodisc (one-level) December 2007	103	6 weeks, 3-, 6-, 12-, 18-, and 24-months	Assessment criteria: reported as “bridging bone” Result at 24 mos: 3/98 (3.0%)	Assessment criteria: radiolucencies > 25% around implant Result at 24 mos: 0/98 (0.0%)	Assessment criteria: height decrease > 3 mm (baseline unclear) Result at 24 mos: 0/98 (0.0%)	Assessment criteria: device motion > 3 mm Migration at 24 mos: 0/98 (0.0%) Subsidence at 24 mos: 0/98 (0.0%)	NR
Bryan (one-level) May 2009	242	6 weeks, 3-, 6-, 12-, 24-months and biennially thereafter	Assessment criteria: reported as “bridging bone” Result at 24 mos: 0/154 (0.0%)	Assessment criteria: NR Result at 24 mos: 0/154 (0.0%)	Assessment criteria: maintenance of height (height change ≤ 2 mm from 3-month post-op height) Reported as part of radiographic success	Assessment criteria: Subsidence success defined as ≤ 2 mm decrease from the 3-month measurement Reported as part of Radiographic Success	Assessment criteria: FSU height/subsidence success based on a patient not having a surgical intervention related to a failure finding for either FSU or subsidence Success at 24 mos: 79.6%
Secure-C (one-level) September 2012	236 (148 randomized, 88 training)	6 weeks, 3-, 6-, 12-, 24-months and annually thereafter	Assessment criteria: Graded 0–IV per Mehren classification Result at 24 mos: Grade 0: 51/198 (25.8%) Grade I: 40/198 (20.2%) Grade II: 69/198 (34.8%) Grade III: 25/198 (12.6%) Grade IV: 13/198 (6.6%)	Assessment criteria: Radiolucencies > 25% around implant Result at 24 months: 0 (0.0%)	Assessment criteria: height change > 2 mm from pre-op baseline Result at 24 mos: 89/189 (47.1%)	Assessment criteria: device motion > 3 mm Migration/displacement/subsidence at 24 mos: 0 (0.0%)	NR
PCM (one-level) October 2012	289 (214 randomized, 75 training)	6 weeks, 3-, 6-, 12-, 24-months and annually thereafter	Assessment criteria: Graded 0–IV per Mehren classification Result at 24 mos: Grade 0: 141/233 (60.5%) Grade I: 34/233 (14.6%) Grade II: 48/233 (20.6%) Grade III: 7/233 (3.0%) Grade IV: 3/233 (1.3%)	Assessment criteria: Radiolucencies > 50% length of prosthesis Result at 24 mos: 14/182 (7.7%; all superior) *AE reports* Occurance at 24 mos: 3/214 patients (1.4%)	Assessment criteria: maintenance of height (≥ 80% of post-op baseline) Result at 24 mos: 160/177 (90.4%)	Assessment criteria: NR *AE reports* Subsidence at 24 mos: 1/214 patients (0.5%) Displacement/Loosening at 24 + mos: 10/214 patients (4.7%)	Assessment criteria: lack of evidence of continuous bridging bone between the adjacent endplates of the involved motion segment or > 2° of segmental movement on lateral flexion/extension radiographs Success at 24 mos: 178/180 (98.9%)
Mobi-C (one-level) August 2013	179 (164 randomized, 15 training)	6 weeks, 3-, 6-, 12-, 18-, 24-months and annually thereafter	Assessment criteria: Graded 0–IV per Mehren classification Result at 24 mos: Grade 0: 14/164 (8.5%) Grade I: 13/164 (7.9%) Grade II: 109/164 (66.5%) Grade III: 16/164 (9.8%) Grade IV: 10/164 (6.1%) *AE reports* Occurance at 24 mos: 9/179 patients (5.0%), 10 events Occurance at 60 mos: 13/179 patients (7.3%), 16 events	Assessment criteria: categorized as none, mild (< 25%), moderate (25–50%), or severe (> 50%) Result 24 mos: mild radiolucency in 2 (1.3%)	Assessment criteria: height change from post-op (at discharge) height Result at 24 mos (randomized cohort): − 0.41 mm ± 0.427 mm Result at 24 mos (training cohort): − 0.45 mm ± 0.336 mm	Assessment criteria: device motion ≥ 3 mm (cranial/caudal for subsidence, anterior/posterior for migration) Subsidence at 24 mos: 0 Migration at 24 mos: 0	Assessment criteria (radiographic failure): evidence of continuous bridging bone and < 2° total angular motion (from flexion to extension) Reported as a part of overall success/failure
Mobi-C (two-level) August 2013	234 (225 randomized, 9 training)	6 weeks, 3-, 6-, 12-, 18-, 24-months and annually thereafter	Assessment criteria: Graded 0–IV per Mehren classification Result at 24 mos: Grade 0: Sup. 23/225 (10.2%); Inf. 20/225 (8.9%) Grade I: Sup. 15/225 (6.7%); Inf. 7/225 (3.1%) Grade II: Sup. 161/225 (71.6%); Inf. 162/225 (72.0%) Grade III: Sup. 17/225 (7.6%); Inf. 17/225 (7.6%) Grade IV: Sup. 8/225 (3.6%); Inf. 6/225 (2.7%) *AE reports* Occurance at 24 mos: 6/234 patients (2.6%), 6 events Occurance at 60 mos: 13/234 patients (5.6%), 13 events	Assessment criteria: categorized as none, mild (< 25%), moderate (25–50%), or severe (> 50%) Result at 24 mos: mild radiolucency in 2 (1.3%)	Assessment criteria: height change from 6-week post-op height Result at 24 mos (superior): − 0.43 ± 0.398 mm Result at 24 mos (inferior): − 0.35 ± 0.385 mm	Assessment criteria: device motion ≥ 3 mm (cranial/caudal for subsidence, anterior/posterior for migration) Subsidence at 24 mos: 0 Migration at 24 mos: 1	Assessment criteria (radiographic failure): evidence of continuous bridging bone and < 2° total angular motion (from flexion to extension) Reported as a part of overall success/failure
Prestige LP (one-level) July 2014	280	6 weeks, 3-, 6-, 12-, 24-months and annually thereafter	Assessment criteria: reported as bone bridging (criteria comparable to Class IV HO assessment per Mehren) Evidence of bridging at 24 mos: 16/280 (5.9%) *AE reports* Occurance at 24 mos: 27/280 subjects (9.6%), 31 events	Assessment criteria: NR Reported as part of Radiographic Success	Assessment criteria: maintenance of height (height change ≤ 2 mm from 6-week post-op height) Result at 24 mos: 205/224 (91.5%)	NR	Assessment criteria: evidence of bone spanning the two vertebral bodies, existence of angular motion stability < 4°, and no radiolucent lines covering more than 50% of the implant surface Success at 24 mos: 219/316 (69.3%)
Prestige LP (two-level) July 2016	209	6 weeks, 3-, 6-, 12-, 24-months and annually thereafter until last subject enrollment (36-, 60-, 84-, and 120- months)	Assessment criteria: Graded 0–IV per Mehren classification Result at 24 mos: Grade 0: Sup. 143/198 (72.2%); Inf. 126/198 (63.6%) Grade I: Sup. 10/198 (5.1%); Inf. 11/198 (5.6%) Grade II: Sup. 13/198 (6.6%); Inf. 22/198 (11.1%) Grade III: Sup. 28/198 (14.1%); Inf. 33/198 (16.7%) Grade IV: Sup. 4/198 (2.0%); Inf. 6/198 (3.0%) Bone bridging comparable to Class IV HO also reported *AE reports* Occurance ≤ 24 months: 22/209 subjects (10.5%), 27 events Occurance ≤ 84 months: 33/209 subjects (15.8%), 40 events	Assessment criteria: NR Reported as part of Radiographic Success	Assessment criteria: maintenance of height (height change ≤ 2 mm from 6-week post-op height at both treated levels) Result at 24 mos: 159/170 (93.5%)	*AE reports* Displacement: ≤ 24 Months: 6/209 subjects (2.9%), 6 events ≤ 84 Months: 7/209 subjects (3.3%), 7 events Subsidence: ≤ 24 Months: 2/209 subjects (1.0%), 3 events ≤ 84 Months: 3/209 subjects (1.4%), 4 events Loosening: ≤ 24 Months: 0/209 subjects (0.0%) ≤ 84 Months: 0/209 subjects (0.0%)	Assessment criteria: angular motion on lateral flexion/extension radiographs > 4° but ≤ 20° and no radiographic evidence of bridging trabecular bone that forms a continuous bony connection with the vertebral bodies (i.e., no bridging bone) at both treated levels Success at 24 mos (superior): 137/197 (69.5%) Success at 24 mos (inferior): 126/195 (64.6%) Success at 24 mos (both levels): 100/196 (51.0%)
M6-C (one-level) February 2019	160	6 weeks, 3-, 6-, 12-, 24-months and annually thereafter	Assessment criteria: Graded 0-IV per Mehren classification Result at 24 mos: Grade 0: 61/150 (40.7%) Grade I: 22/150 (14.7%) Grade II: 50/150 (33.3%) Grade III: 16/150 (10.7%) Grade IV: 1/150 (0.7%)	Assessment criteria: categorized as none, mild (< 25%), moderate (25–50%), or severe (> 50%) Result at 24 mos: None: 144 (96.0%) Mild (< 25%): 6 (4.0%) Moderate (25–50%): 0 (0.0%) Severe (> 50%): 0 (0.0%)	Assessment criteria: disc height comparison from pre-op height Height preoperatively: 3.22 ± 0.73 mm Height at 24 months: 5.31 ± 1.02 mm	Assessment criteria: NR Loosening at 24 mos: 4/150 (2.6%) Subsidence at 24 mos: 1/150 (0.7%) Migration at 24 mos: 0/150 (0.0%)	NR
Simplify (one-level) September 2020	166 (150 randomized, 16 training)	6 weeks, 3-, 6-, 12-, 24-months and annually thereafter	Assessment criteria: Graded 0–IV per Mehren classification Results at 24 mos: Grade 0: 20/139 (14%) Grade I: 16/139 (12%) Grade II: 80/139 (58%) Grade III: 11/139 (8%) Grade IV: 10/139 (7%)	NR	Assessment criteria: disc height comparison from pre-op height Height preoperatively: 3.31 ± 0.74 Height at 24 months: 4.24 ± 0.94 mm Superior and inferior adjacent level height also reported	Assessment criteria: device motion > 3 mm compared to position at implantation Migration at 24 months: 0 *AE reports* Implant collapse or Subsidence through Day 790: 0	NR
Simplify (two-level) April 2021	200 (182 randomized, 18 training)	6 weeks, 3-, 6-, 12-, 24-, 36-months and annually thereafter	Assessment criteria: Graded 0–IV per Mehren classification Results at 24 mos: Grade 0: 21/166 (13%) Grade I: 21/166 (13%) Grade II: 106/166 (64%) Grade III: 8/166 (5%) Grade IV: 8/166 (5%)	NR	Assessment criteria: height change from post-op height Result at 24 months: Superior component: 1.19 ± 0.81 mm Inferior component: 0.84 ± 0.96 mm Superior and inferior adjacent level height also reported Assessment criteria for disc height success also reported but with no corresponding results	Assessment criteria: device motion > 3 mm compared to position at implantation Migration at 24 months: 0 *AE reports* Implant collapse or Subsidence through Day 790: 0	NR

Other radiographic outcomes reported focused on ROM, non-union/fusion failure. Reported on 24 month outcomes but some had longer

*NR* Not reported, *AE* Adverse event

Using the proposed classification system, we observed reasonable agreement among experienced investigators. Previous researchers have employed classification systems for anterior bone loss around cTDRs [28, 29], rather than assessing the anterior, centralized, and posterior regions of the endplates as we proposed in the present study. In addition, previous investigators did not assess the repeatability or reproducibility of their classification systems for anterior bone loss, making it difficult to compare prior research with the present study. Chen and colleagues [29] evaluated radiographs of the Bryan disc for anterior bone loss, with a grade ranging from 0, with no bone loss, to 2 for “obvious bone regression.” They found that anterior bone loss with a Grade of 1 or 2 occurred in the inferior endplate of 54/121 patients (44.6%) from their series within the first 6–12 months after surgery [29]. Kaiser et al. [28] performed radiographic measurements of the relative distance of anterior bony coverage by the endplates of three different cTDR designs over time. The researchers assessed the anterior bone loss as minor (< 5%), moderate (5–10%), and severe (> 10%). Among the 156 cTDR evaluated, Kaiser [28] found anterior bone loss in 57.1% of cases evaluated. In our study, we found that binary radiographic assessments, such as the presence of absence of bone loss in a particular location, as well as subsidence, migration, and progressive core height loss, were found to exhibit the highest agreement among investigators. Lower agreement was found in our study among investigators when grading the severity of bone loss, indicating the lack of consensus as to what constitutes mild, moderate, or severe bone loss. For example, Kaiser [28] considered 10% of anterior bone loss to be severe, whereas the investigators in the present study considered loss of 1/3 of the endplate coverage to be severe. With regard to the accuracy of the proposed classification system, we expect that broad use of the tool by experts will help establish “gold standard” classifications which can be used to assess validity in future work. Our study confirms the utility of the HO classification system proposed by McAfee et al. [18] Because HO can occur in some regions of a cTDR that also are undergoing bone loss, we consider it important to include HO assessment in the characterization of bony changes using radiographs.

We would like to underscore several limitations for the reader. In the present study, we focused on developing a classification system for plain radiographs because of their ubiquity in clinical follow up of spine patients. However, plain radiographs can underestimate bone loss. Variations in imaging technique, such as tilting or obliquity of the cTDR with respect to the plane of radiographic study, greatly complicate the assessment of the bone-implant interface. Differences in radiographic exposure over time can also confound the interpretation of the bone-implant interface, if insufficient or inconsistent beam penetration of the bone occurs over time. Additionally, the presence of bony or radiographic abnormalities can also affect the interpretation of the radiographs. These limitations may help explain, in part, the lower agreement in the assessment of bone loss severity among investigators, and lower-quality radiographs may complicate use of the classification system in practice. In addition, plain radiographs have limitations in diagnosis of the underlying causes of the bone loss, and whether the mechanism in a particular patient is due to a septic or aseptic etiology [25]. Furthermore, it can be challenging to assess whether loosening is caused by failure to achieve initial stable fixation of the metallic endplates. Although the progression of radiolucencies can be useful in this regard, subtle bone-implant relative motion may also be appreciated by alternating flexion and extension views while the images are stabilized on the superior and/or inferior vertebral body. We did not consider such flexion–extension image assessment as part of the present study. We similarly did not consider AP view radiographs, which restricted our ability to clearly explore changes occurring lateral to the implants. Despite these limitations, due to the prevalence of radiographs in cTDR patient care, improving the consistency in terminology and classification of radiographic changes at the bone-implant interface is expected to improve communication among clinicians.

## Conclusions

In summary, a standardized nomenclature for bony changes following cTDR will facilitate accurate and reproducible scientific communications regarding the clinical outcomes of this procedure. The novel system proposed demonstrated good concordance among experienced investigators in this field and represents a useful advancement for improving reporting in cTDR studies.

### Supplementary Information

**Additional file 1: Fig. S1.** Examples of endplate rounding. **Fig. S2.** Examples of cystic erosion with diffuse margin adjacent to endplate. **Fig. S3.** Examples of cystic erosion with sclerotic margin adjacent to endplate. **Fig. S4.** Example of cystic erosion (red arrow) along with endplate rounding (yellow arrow).
