# Supplementary material for: Proposal for a classification system of radiographic bone changes after cervical disc replacement
Source: J Orthop Surg Res. 2024 Apr 3;19:218. doi: 10.1186/s13018-024-04679-y (PMC10988897; doi:10.1186/s13018-024-04679-y)
Supplement: Supplementary file 1 — Additional file 1: Fig. S1. Examples of endplate rounding. Fig. S2. Examples of cystic erosion with diffuse margin adjacent to endplate. Fig. S3. Examples of cystic erosion with sclerotic margin adjacent to endplate. Fig. S4. Example of cystic erosion (red arrow) along with endplate rounding (yellow arrow). [file 13018_2024_4679_MOESM1_ESM.docx]

Supplementary Material: Examples of Endplate Rounding


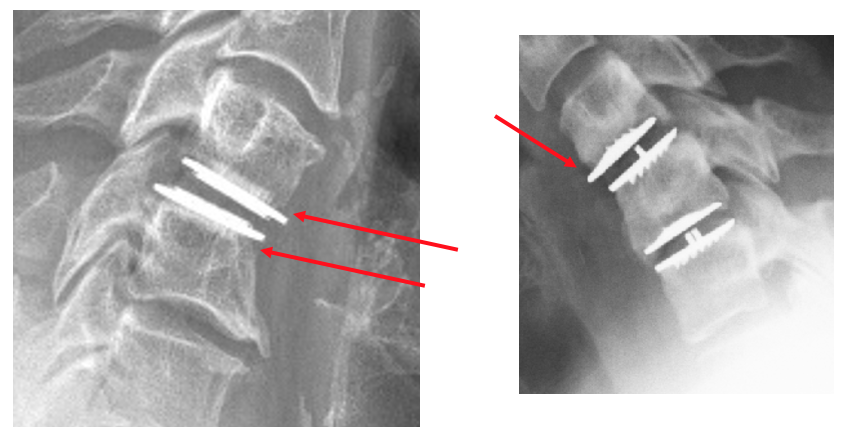


Figure S1: Examples of endplate rounding.


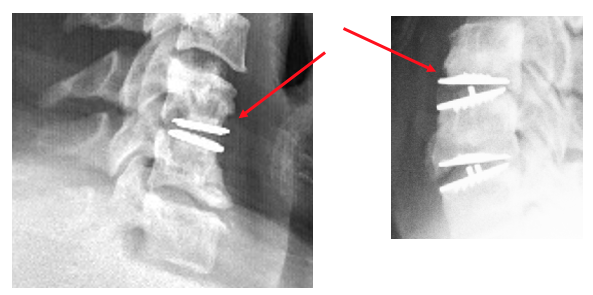


Figure S2: Examples of cystic erosion with diffuse margin adjacent to endplate.


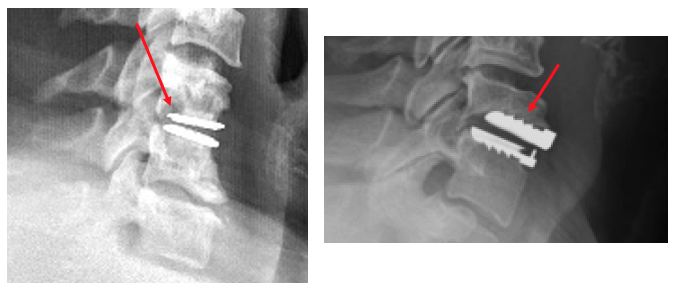


Figure S3: Examples of cystic erosion with sclerotic margin adjacent to endplate.


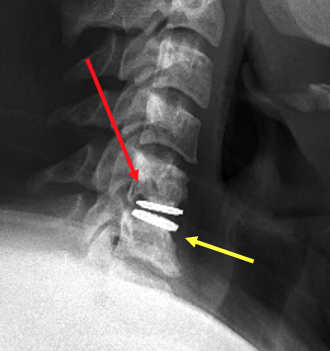


Figure S4: Example of cystic erosion (red arrow) along with endplate rounding (yellow arrow).
